# Protein–Protein Interaction Hotspots Carved into Sequences

**DOI:** 10.1371/journal.pcbi.0030119

**Published:** 2007-07-13

**Authors:** Yanay Ofran, Burkhard Rost

**Affiliations:** 1 Department of Biochemistry and Molecular Biophysics, Columbia University, New York, New York, United States of America; 2 Columbia University Center for Computational Biology and Bioinformatics (C2B2), New York, New York, United States of America; 3 NorthEast Structural Genomics Consortium (NESG), Columbia University, New York, New York, United States of America; Spanish National Cancer Research Centre (CNIO), Spain

## Abstract

Protein–protein interactions, a key to almost any biological process, are mediated by molecular mechanisms that are not entirely clear. The study of these mechanisms often focuses on all residues at protein–protein interfaces. However, only a small subset of all interface residues is actually essential for recognition or binding. Commonly referred to as “hotspots,” these essential residues are defined as residues that impede protein–protein interactions if mutated. While no in silico tool identifies hotspots in unbound chains, numerous prediction methods were designed to identify all the residues in a protein that are likely to be a part of protein–protein interfaces. These methods typically identify successfully only a small fraction of all interface residues. Here, we analyzed the hypothesis that the two subsets correspond (i.e., that in silico methods may predict few residues because they preferentially predict hotspots). We demonstrate that this is indeed the case and that we can therefore predict directly from the sequence of a single protein which residues are interaction hotspots (without knowledge of the interaction partner). Our results suggested that most protein complexes are stabilized by similar basic principles. The ability to accurately and efficiently identify hotspots from sequence enables the annotation and analysis of protein–protein interaction hotspots in entire organisms and thus may benefit function prediction and drug development. The server for prediction is available at http://www.rostlab.org/services/isis.

## Author Summary

Interactions between proteins underlie all biological processes. Hence, to fully understand or to control biological processes we need to unravel the principles of protein interactions. The quest for these principles has focused predominantly on the entire interfaces between two interacting proteins. However, it has been shown that only few of the interface residues are essential for the recognition and binding to other proteins. The identification of these residues, commonly referred to as binding “hotspots,” is a first step toward understanding the function of proteins and studying their interactions. Experimentally, hotspots could be identified by mutating single residues—an expensive and laborious procedure that is not applicable on a large scale. Here, we show that it is possible to identify protein interaction hotspots computationally on a large scale based on the amino acid sequence of a single protein, without requiring the knowledge of its interaction partner. Our results suggest that most protein complexes are stabilized by similar basic principles. The ability to accurately and efficiently identify hotspots from sequence enables the annotation and analysis of protein–protein interaction hotspots in an entire organism and thus may benefit function prediction and drug development.

## Introduction

Interactions of proteins are at the heart of almost every biological process. Thus, the understanding of biological mechanisms requires the knowledge of protein–protein interactions and the molecular principles that underlie them. Large-scale studies unravel networks of protein–protein interactions in cells and identify interacting pairs of proteins [1–5]. However, to fully understand these interactions, and to manipulate them, we need to identify the residues that account for binding of the proteins and stabilizing the complexes. It has been postulated that only very few of the residues in protein–protein interfaces are absolutely essential for the interaction (in a typical 1,200- to 2,000-Å^2^ interface, less than 5% of interface residues contribute more than 2 kcal/mol to binding. In small interfaces, this can mean as few as one amino acid on each protein) [6]. These residues may be instrumental in understanding the interaction and could be desired drug targets [7].

The ability to predict hotspots on a large scale may assist in identifying, analyzing, and comparing binding sites for drugs. Given a detailed 3-D structure of a complex, the residues crucial for binding are often identifiable. The Hendrickson lab, for instance, identified the most essential binding residues from their 3-D structure of HIV glycoprotein (gp120) and CD4 receptor [8]. Unfortunately, 3-D structures are available for less than 1% of all known pairs of interacting proteins. In the absence of 3-D structures, the most conclusive way to probe the importance of particular residues for interaction is to experimentally mutate them, typically to alanine, and measure the effect of this substitution on the interaction [9,10]. Many experiments have demonstrated that most interface residues could be mutated without affecting the affinity of the protein to its partners [11,12]. Those few residues that, upon mutation, change the affinity are often assumed to be the most essential for the interaction and are deemed “hotspots” [6]. The limited overlap between interface residues and hotspots is demonstrated in Figure 1, which depicts the complex of the human growth hormone and its receptor [13]. In the bound state (Figure 1A), a large patch on the surface of the receptor is buried in the interface. There are 31 residues on the receptor that are in physical contact with a hormone (Figure 1B). However, mutation experiments indicate that only six of these residues are energetically crucial for the interaction (Figure 1B).

**Figure 1:**
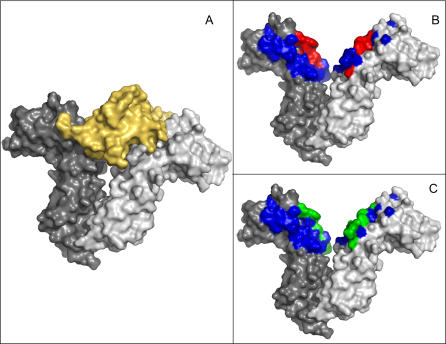
Protein–Protein Interfaces, Hotspots, and Predictions Residues that are part of protein–protein interfaces often constitute a large fraction of the protein. Hotspot residues, namely residues that upon mutation hamper the interaction, are only a small fraction of these interface residues. Interestingly, methods designed to predict interface residues usually capture only a small fraction of them. (A) Human growth hormone (yellow) bound to the extracellular portion of its homodimeric receptor. (B) The chains of the receptor (gray) are 201 residues long. The protein–protein interface covers 31 of these residues (blue and red) on each of the chains. Mutating one of the six residues colored in red abrogates or severely hampers the interaction. (C) A prediction method (ISIS; see text) that was designed to identify all interface residues managed to capture only five of the interface residues (colored green).

The ways to identify hotspots have been subject to theoretical debates. It has been pointed out that given the structural and physicochemical complexity of proteins, the physicochemical features of a protein are not a simple sum of the features of its individual residues [14]. Therefore, single mutations may not always convey accurate assessments of the contribution of a residue to the interaction [15,16]. The theoretical validity of this argument notwithstanding, alanine scans have become the most widely used tool for identifying binding sites. While single mutations may not be tantamount to isolating the contribution of a single residue to the interaction, they are still considered a good approximation. Here, we adopt the following operational definition: if a mutation of a residue in a protein–protein interface changes the binding energy of the protein to its binding partner substantially (ΔΔG > 2.5 kcal/mol), then this residue is a hotspot residue.

To the best of our knowledge, there is currently no method that was designed to identify hotspots from sequence. However, many methods attempt to use sequence or structure to identify which residues are located in the interface between proteins [17–32]. Many of the methods that identify residues in protein–protein interfaces reach impressive levels of *positive accuracy* (residues correctly predicted to be in protein–protein interfaces as a fraction of all residues predicted to be in protein–protein interfaces; often also referred to as *selectivity,* or *precision;* Equation 1). However, their *coverage* (residues correctly predicted in interfaces as percentage of observed interface residues; often also referred to as *sensitivity,* or *recall;* Equation 2) remains fairly low. In other words, although these methods attempt to identify all interface residues (all the residues that are colored blue or red in Figure 1B), they capture only a small fraction of them (e.g., only the green residues in Figure 1C). We hypothesized that the reason for the low coverage of many prediction methods might be that the residues that are missed are more similar to the general population of surface residues than to the essential residues (i.e., they are inconsequential for the interaction). Therefore, a machine-learning algorithm trained on all protein–protein interface residues may learn to disregard the non-hotspot residues as noise, and identify only hotspot residues as the signal to be learned.

To test this hypothesis, we applied ISIS, a prediction method developed for the prediction of all interface residues [28], to the task of predicting only hotspots. ISIS was never trained on hotspots (Methods). Instead, we trained on *all* interface residues found in Protein Data Bank (PDB) complexes (i.e., all interface residues were labeled “positive,” and all other residues were labeled “negative”). The features on which ISIS was trained included the sequence environment of each residue (four residues on each side), the evolutionary profile of all nine residues in that window, the predicted solvent accessibility of the residue and the solvent accessibility of its immediate sequence environment (one residue on each side), the predicted secondary structure state of the residue and its immediate sequence environment (one residue on each side), and a conservation score for each residue. Like several other methods mentioned above, ISIS predicts residues in protein–protein interfaces very accurately (∼90% accuracy). However, at this high level of accuracy, ISIS identifies fewer than 5% of the residues that were experimentally mapped to the interface.

The novelty here is that we applied a generic interface-prediction method to the specific task of identifying only the residues that are crucial for stabilizing the interactions (i.e., the hotspots). The results demonstrated a surprising overlap between two principally unrelated datasets, namely on the one hand the subset of residues that was identified by experimental alanine mutations as hotspots, and on the other hand the subset of residues predicted by ISIS to be protein–protein interface residues. We obtained a large dataset of hotspots that were determined experimentally through alanine scans (Methods) and assessed the performance of ISIS on these hotspots. The results confirmed our hypothesis that the residues predicted by the machine-learning method are, in fact, the hotspots. Analysis of the results indicated that accurate predictions of hotspots required the combination of sequence features, evolutionary information, and predicted structural features; all this information was generated from the amino acid sequence, suggesting that the commonalities of hotspots have been imprinted clearly onto amino acid sequences in the course of evolution.

## Results

Using 296 point mutations from 30 proteins, we compared the residues predicted by ISIS with the ones experimentally identified to be hotspots (Methods). We first analyzed the results for two representative examples. Then, we assessed the performance in predicting hotspots based on the analysis of the entire dataset of 296 mutations. Note that although the 3-D structures for most of these proteins were experimentally known, ISIS predicted interface residues from sequence alone. At no stage of the predictions did we use the experimentally determined structure. The only way in which we used 3-D information was to visualize our results, as we mapped the predictions to the experimentally determined structure (Figure 2).

**Figure 2:**
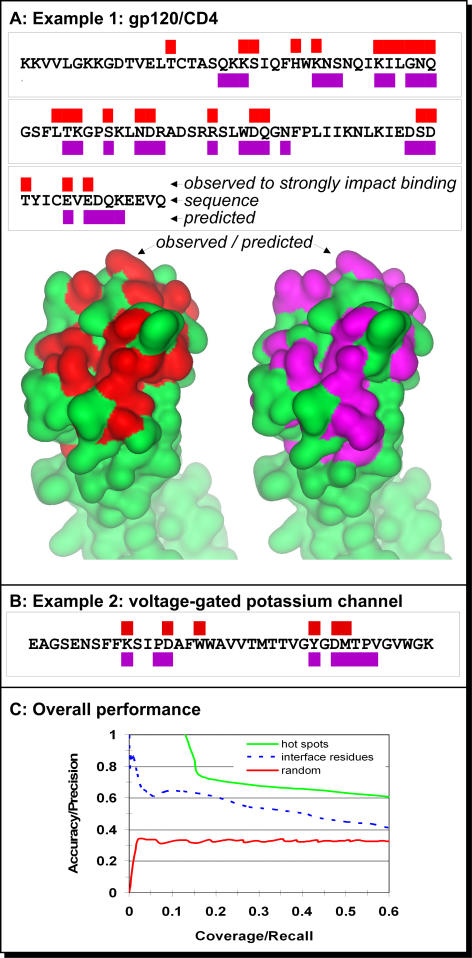
Accuracy of Prediction of Hotspots The ability to identify the residues that account for most of the energy of binding is assessed both on particular proteins and on a large dataset of alanine scans. (A) Alanine scans and predictions of essential interface residues in the V1 domain of CD4. The red rectangles (above sequence) mark positions that were shown to have significant effect on the affinity of the binding between CD4 to gp120 upon substitution to alanine [33]; the same residues are colored in red on the lower left surface representation of CD4 (PDB ID 1wiq_A). The green rectangles (below sequence) mark positions predicted to participate in a protein–protein interaction; these residues are also colored in violet on the lower right. Note that five of the residues predicted in interfaces were not mutated in the alanine scan. Thus, we cannot evaluate their correctness and left them out of this analysis. (B) Hotspots experimentally observed and predicted for the shaker voltage-gated potassium channel. All predictions and experimental substitutions [34] for this stretch are reported in this figure. (C) Accuracy versus coverage in predicting hotspots and interface residues. The performance of ISIS (green) and random assignment (red) using 296 alanine scans as gold standard. The data were compiled for a set of proteins that was not used for developing the method. The stronger the confidence in our prediction, the higher the accuracy and the lower the coverage (i.e., when we select the strongest predictions [moving upward in the figure], most of these are correct). With an accuracy of approximately 0.61 (righthand side of the plot), ISIS correctly predicted most of the interacting residues in our test set. The performance of ISIS in the task for which it was originally developed, namely predicting all interface residues (broken blue), is substantially worse than its performance on hotspots.

### HIV gp120/CD4 Receptor Complex

One of the most comprehensive alanine scans of all the complexes with known 3-D structures is that between the CD4 receptor and the HIV glycoprotein gp120. This interaction involves backbone interactions, mainly on the gp120 side. However, we focused our analysis on the human CD4 receptor. Ashkenazi et al. [33] sequentially mutated many residues in the V1 domain of the CD4 receptor and studied the effect of each substitution on the binding affinity between CD4 and the HIV gp120 protein. Using a set of specific antibodies, they also assessed which mutation had no effect on the structure. They identified 25 positions within a stretch of 94 residues on CD4 that upon substitution changed the affinity of CD4 substantially, without strongly altering the conformation of the protein. Within the same 94-residue segment (Figure 2A), we predicted 30 residues as interface residues; 19 of these were found experimentally to have a strong effect on binding. Of the six residues that ISIS missed, four were next to predicted interface residues. Five of the predictions that were not confirmed experimentally were residues that were not mutated in the study. Our method uses predicted structural features (solvent accessibility and secondary structure). Hence, its performance depends to some extent on the accuracy of these predictions. If we have a 3-D structure of the unbound chain, we can improve accuracy and coverage by using the experimental rather than the predicted features. For example, when we used the unbound structure of CD4 as input for ISIS, we found a few additional residues that were not identified from sequence alone. The two residues that scored highest (i.e., about which we were most confident that they participate in binding) were Arg59 and Phe43. The high-resolution structure of the complex between gp120 and CD4 complex [8] revealed two residues as the most important contacts between these two proteins: Arg59 and Phe43.

### Voltage-Gated Potassium Channel

For a variety of reasons, membrane proteins are a particularly popular target for alanine scans. One such alanine scan is available for the shaker voltage-gated K^+^ channel [34]. Within a region of 29 consecutive residues that have been scanned, eight have a significant effect on the affinity of the channel to its inhibitors agitoxin2 and charybdotoxin. We used this region as input to our method, ignoring any available structural information, and predicted 13 residues (Figure 2B). Seven of the eight residues that were found experimentally were predicted by ISIS; the only residue that was missed is buried in the structure and hence is likely to affect the interaction indirectly through a conformational change. Of the six residues in our prediction that did not coincide with the residues implicated as important by the alanine scanning, five coincided with positions that were found to have significant although less dramatic effects on binding [34].

### Performance over Entire Dataset

Within our set of alanine scans, almost all binding residues predicted by ISIS were found experimentally to have significant effect on binding (Figure 2C). Furthermore, more than 90% of the negative predictions (predicted not to be involved in protein–protein interactions) were confirmed experimentally to have no effect on the energy of binding. These results were particularly surprising in light of the fact that ISIS never explicitly evaluated any energetic parameters. Using different confidence thresholds (i.e., picking a different point on the curve in Figure 2C), it is possible to increase accuracy (true positives/all positives) at the expense of coverage (true positives/predicted positives). Note that the results for the two examples (Figure 2A and 2B) discussed in detail are similar to the performance of ISIS on the entire dataset of 296 mutations.

## Discussion

### Hotspots Are Easy To Identify but Hard To Define

We used ISIS to represent methods that predict interface residues at high accuracy and low coverage. The results suggested that the system of neural networks that underlies ISIS learned to identify the hotspots, despite the fact that they were only a small subset of the samples that were labeled as interaction residues. The system effectively disregarded most of the residues observed in interface (i.e., the pupil [neural network] clearly ignored the teacher [labeled data]). We found that the residues ignored were mostly non-hotspot residues. These results indicated that the biophysical common denominators of hotspots are so pronounced that the neural networks could identify them without specific labeling in the training phase.

What are these features that are common to hotspots? Unfortunately, we cannot simply list a few rules or features to describe these commonalities. The neural networks identified a set of complex nonlinear correlations between the input features we used and hotspot residues. It is impossible to translate the subtle and complex dependencies that were identified by the neural networks into simple explanations, or a set of rules, in English. However, it is possible to infer which features are more or less relevant. To that end, we trained several systems using different combinations of input features. Neural networks that were trained only on the sequence environment of interface residues performed only slightly (although significantly) better than random (unpublished data). Adding evolutionary information significantly improved performance on both interface residues and on hotspots. This result was somewhat surprising given that the conservation of predicted hotspots was only marginally different from that of all other residues (Figure 3). Conversely, predicted non-hotspot residues were only marginally less conserved than the background. In other words, although the overall difference in conservation was marginal, the addition of this information to the neural network input substantially improved performance. Apparently, the neural networks have learned to distinguish between conservation that is indicative of hotspots, and conservation that is not. Strikingly, they did so without being trained on hotspots. This underscores why linear combinations of input features did not suffice and why the extraction of singly important commonalities would at best be misleading.

**Figure 3:**
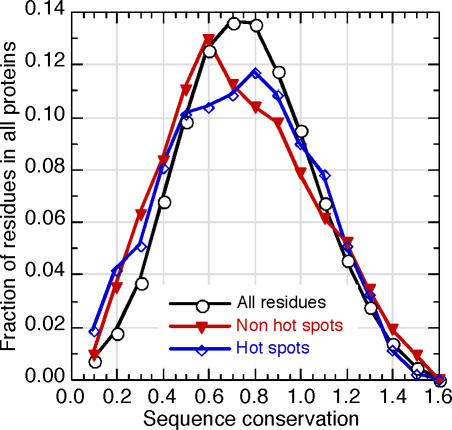
The Common Features of Hotspots Are Hard To Identify without Machine Learning Physicochemical, structural, and evolutionary features differentiate hotspots from other residues. However, while each of these features is crucial for the success of the prediction, a simple, linear combination of them will not suffice. The distributions of residue conservation (*x*-axis, HSSP [46] conservation score) are compared between the entire sequences of the proteins in the dataset (black circles), hotspots (blue diamonds), and residues with no effect (measured by alanine scans) on protein–protein binding (red triangles). The *y-*axis gives the fraction of residues with a given level of conservation. The differences are marginal, but the overall effect of conservation on the prediction is substantial.

The analysis of the contribution of each feature suggested that successful predictions of hotspots required the combination of all features. However, even when some of these features were not available, ISIS still could provide accurate predictions (e.g., 15% of the proteins found less than ten homologues in today's databases). For these proteins, the success in predicting hotspots was lower, but still significantly higher than random (at 70% positive accuracy, >10% of the experimentally determined hotspots were identified compared with about 70%/20% for all proteins; Figure 2).

### Successful Hotspot Predictions Require Specific In Silico Tools

We did not benchmark the ability of prediction methods other than ISIS to predict hotspots. The main reason was that no existing method (including ISIS) was designed to predict hotspots. The ability of ISIS to identify hotspots is an unintended consequence of the power of neural networks. Therefore, when comparing ISIS with other methods, one should remember that this comparison does not benchmark these methods in the task for which they were originally developed. Still, the question remains of whether or not any method designed to predict interface residues could predict hotspots at levels of accuracy as high as the ones we reported for ISIS. To address this question, we applied a few representative interface prediction methods to the task of predicting hotspots. In particular, we chose methods that rely on a different input feature. Analysis of the results indicated that methods that did not rely on a combination of physicochemical features, evolutionary conservation, and structural features failed to identify hotspots.

### What Does It Take To Predict Hotspots?

We applied several prediction methods that were designed to identify interface residues to the task of predicting hotspots. To eschew obfuscation: our aim was not to benchmark methods not designed to identify hotspots. Instead, we applied these methods to narrow down the features needed to successfully predict hotspots.

The evolutionary trace (ET) method [35] correlates evolutionary importance of residues with their importance for function. We used ET to represent the approach that relies predominantly on evolutionary conservation. Gallet et al. [22] have attempted to predict interaction sites from simple biophysical features; the method computes the hydrophobic moment [36] around each residue based on its sequence environment to determine whether this residue could be a binding site. ProMate [26] extracts its input from the 3-D structure of an unbound protein; we used it to represent methods that rely on experimentally determined 3-D structures. We also included another method that predicts interfaces exclusively using amino acid information (and no aspects of predicted structure or evolutionary profiles) [29]. We arbitrarily chose the operating point at which the coverage of hotspots was 15% (Methods) and checked the accuracy of each method for this coverage (Figure 4). ISIS and ProMate, the two methods that were most successful, use physicochemical features, evolution, and structural features. ISIS is the only sequence-based method, and the structural features it uses are based on predictions. ProMate, which relies on the 3-D structure, performed even better. The conclusion of this analysis is that no single feature suffices to characterize hotspots. Rather, it takes a complex combination of the aforementioned features that defines a residue as a hotspot.

**Figure 4:**
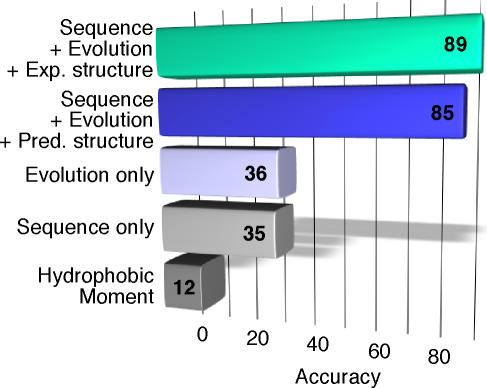
Accuracy of Prediction of Hotspots at Coverage Levels of 15% Several approaches were introduced in the past for the prediction of interface residues. We applied methods that rely on different features to the task of predicting hotspots (to which none of them was optimized). The hydrophobic moment method represented the approach that relies exclusively on local physicochemical factors. The evolutionary approach was represented by the ET method, which relies on conservation to identify functionally important residues. A knowledge-based tool we introduced in the past represented the sequence-only approach. Finally, ProMate, a method for predicting interaction sites from unbound structures, represented the structure-based approach.

### How Hotspots Differ from Other Interface Residues

It is apparent that the neural networks identified some common denominators between hotspots that distinguish them from other interface residues. This question is hard to address given our current gold standard (namely the dataset of experimental alanine scans). The number of features we use for the prediction (189) is greater than the number of positive data points in our set of alanine scans. To determine to what extent each input feature differentiates between hotspots and other interface residues, we need a substantially larger dataset of hotspots and non-hotspot residues. This could be achieved if we assume that ISIS indeed identifies hotspots. Thus, by running ISIS on a large dataset of interface residues, we can create a large dataset of predicted hotspots and a large dataset of interface residues that are predicted not to be hotspots. Then, we can use these large datasets to analyze the characteristics of hotspots versus the characteristics of other interface residues. We did this using the large dataset of interface residues that was used as a test set for training ISIS. On this dataset we compared the residues that were classified by ISIS as positive (i.e., hotspots) with those that are annotated experimentally as interface residues but are classified by ISIS as negatives. Table 1 is based on the multiple sequence alignment of each protein in this dataset. For each interface residue, it shows the average occupancy of its position by each type of amino acid. We also present the average occupancy of each residue in the alignment for experimentally determined hotspots (through alanine scan). These values are presented in parentheses, as the data that underlie them are sparse (only 100 positions). Note that for some amino acids there are significant differences between hotspot and non-hotspot interface residues, while for others there are no substantial differences. Table 1 also presents the *p*-value for the difference based on a *t*-test. Note, for example, the 400% overrepresentation of arginine in predicted hotspots (and the extremely low *p*-value) with reference to other interface residues. However, the percentages of lysine are virtually the same for both categories. Thus, it is not simple considerations of hydrophobicity that characterize hotspots. Four aliphatic residues are depleted in hotspots (A, V, I, and L), while amide side chains are overrepresented (N and Q). However, the role of aromatics is unclear since tyrosine is enriched in hotspots, phenylalanine is depleted, and tryptophan has similar propensities across the interface. The experimental values (shown in parentheses) are very close to the values obtained for the predicted hotspots, supporting our assumption that ISIS identifies hotspots. However, the limited amount of experimental data limits our ability to elaborate on this comparison. We also compared the conservation and the structural features of both groups. As shown in Figure 3, there were hardly any differences in conservation. However, the most striking differences were found between structural features (Table 2). The secondary structure state of 39% of the non-hotspot interface residues was a loop. In the predicted hotspots, on the other hand, 57% of the residues were in a loop state. In both categories, the rest of the residues were divided roughly equally between helices and strands. Again, there is a striking agreement between the properties of predicted hotspots and the properties of experimental hotspots, despite the fact that ISIS was trained on all interface residues. Predicted hotspots were also much more accessible to solvent than other interface residues.

**Table 1:**
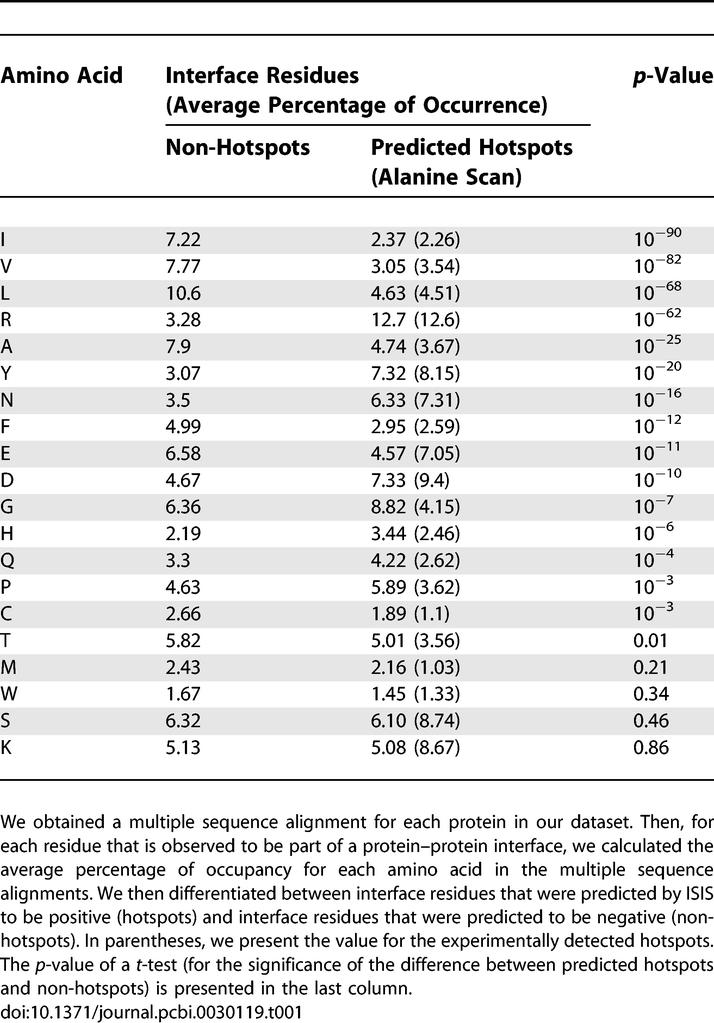
Position Occupancy in Hotspots versus the Rest of the Interface

**Table 2:**
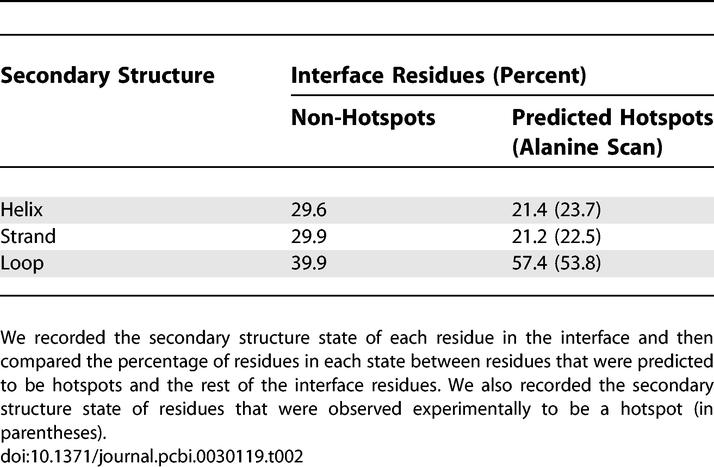
Secondary Structure in Hotspots versus the Rest of the Interface

### Are All Hotspots Similar?

Several studies suggested that hotspots have certain structural characteristics that differentiate them from other residues [37,38]. The Baker lab has shown that given a 3-D structure of a protein complex, it is possible to predict the results of alanine scans specifically and accurately [39,40]. This indicates that alanine scans indeed capture some genuine physicochemical commonalities of interaction hotspots that could be identified by a general method that is applicable to all protein complexes. The in silico alanine scanning is based on analysis of the 3-D structure of the interface between two proteins. Thus, it requires a high-resolution structure of the protein complex, while ISIS needs only sequence of a single chain regardless of its binding partner. On the other hand, in silico alanine scanning produces numerical prediction of the ΔΔG, While ISIS produces a binary prediction (hotspot/non-hotspot). We compared our predictions to those of the in silico alanine scanning by translating their numerical predictions to binary ones according to cutoffs defined above. Of 55 experimental mutations with ΔΔG > 2.5, in silico alanine scanning identified 36 (66%) residues as hotspots. At this coverage, ISIS reached accuracy of about 60% while the in silico alanine scanning reached accuracy of greater than 75%. Scaled to an accuracy of 80%, ISIS identified 18 of these mutations (33%). Thus, for similar levels of positive accuracy, the coverage of ISIS is roughly half that of the in silico alanine scanning. Obviously, when structures of the complex are available, the in silico alanine scan is a powerful tool for identifying hotspots. However, when only the sequence is available, ISIS can provide accurate predictions for a substantial fraction of the hotspots. Our results indicate that some hotspots can be predicted accurately not only without relaying the 3-D structure of the complex but even without the 3-D structure of the unbound proteins. Furthermore, our predictions did not require knowledge of the binding partner. Analyzing a single protein using ISIS typically requires a few minutes. Thus, ISIS may allow large-scale analysis of hotspots at a relatively small CPU cost.

## Methods

### Dataset.

We used the ASEdb database of experimental alanine scans [12], which lists residues that were mutated to alanine and the effect (in terms of ΔΔG) this mutation had on the interaction between two proteins. We checked the correlation between the predictions and the residues that were shown experimentally to substantially affect the affinity of the proteins in a complex to each other. In order to reduce the number of cases in which the effect of the mutation on binding was not due to a change in the interface (e.g., the cases in which the mutation destabilized the structure), we considered only exposed residues in proteins of known structure. Thus our test set included 80 protein chains with hundreds of experimental substitutions. From among these, we analyzed the mutations that substantially changed the binding energy (ΔΔG > 2.5 kcal/mol), and those that had no effect (ΔΔG = 0). Altogether, we attempted to predict the experimental effect of 296 substitutions. The predictions were performed using ISIS [28]. ISIS can take as input either sequence or the coordinate of 3-D structure of unbound chains (the results are more accurate when using known 3-D structures). However, for all values reported here, we ran ISIS from sequence alone.

### Measuring performance.

The accuracy and coverage of ISIS were measured using ratios derived from *TP* (true positives), defined as the number of residues predicted by ISIS (below) to be in a protein–protein interface and observed to be in a hotspot (i.e., was found to have an extreme effect on binding; ΔΔG > 2.5kcal/mol); *FP* (false positives), defined as the number of residues predicted in protein–protein interfaces, were found however, upon mutation, to have no effect on binding (ΔΔG = 0); and *FN* (false negatives; i.e., the number of residues predicted not to be in a protein–protein interface that were observed to have a strong effect on binding [ΔΔG > 2.5 kcal/mol]). We used the following equations:

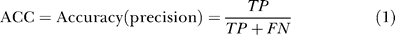


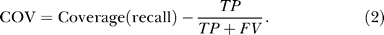



### ISIS.

ISIS is a knowledge-based method we developed to identify interface residues from sequence [28]. It is based on a system of neural networks and uses as input the sequence environment of each residue, its evolutionary profile (the frequency of each type of amino acid in a given position of the alignment), and its predicted secondary structure and accessibility to the solvent. In particular, when a sequence is submitted as a query, ISIS runs PSI-BLAST [41], generates a multiple sequence alignment, and produces an evolutionary profile for each residue. These data are then sent to PROF [42], a system of neural networks that predicts the secondary structure state and the solvent accessibility of each residue. Finally, the sequence environment, the evolutionary profile, and the predicted structural features serve as input to another neural network, which annotates each residue as interface or noninterface. ISIS was trained on a nonredundant version of all transient protein–protein interfaces [27] in the PDB. (The 3-D structures were used only to identify the residues spatially in the interface. No experimental 3-D information was used for training.)

### Training the neural network: First-level prediction.

We trained standard feed-forward neural networks with back-propagation and momentum terms on windows of nine consecutive residues. A window was defined as positive if the central residue had any atom that was within 6 Å of any atom in a different protein. This yielded a set with 59,559 positive samples. We trained on two-thirds of the data and tested it on the remaining one-third.

### Second-level refinement filter.

Next, we filtered the raw network predictions. Our analysis of protein interfaces at the sequence level suggested that most interacting residues have other interacting residues in their sequence neighborhood. Therefore, we eliminated predictions with fewer than seven raw predictions within ten adjacent residues (five on either side).

### Random model.

To obtain the expected coverage and accuracy at random, we reshuffled the predictions in the following way: each protein was represented by two strings of the same length, one representing its sequence and the other representing the predictions (“P” for an interacting residue, “–” for a noninteracting residue). Then, we split the prediction string in half and assigned the predictions of the first half of the sequence to the second and vice versa. This process accounted for any size effect that could be caused by the number of predictions and for any effect caused by the heterogeneous distribution of contacting residues along the sequence. Furthermore, it enabled us to find a specific expectation for each scaling of the prediction. We generated different random models for different values of the receiver operating characteristic (ROC)–like curve (Figure 2C). Our background model captured how random our predictions were rather than how well we could predict interface residues at random.

### No overlap between datasets used for development and for assessment.

ISIS was developed on a dataset of 1,134 chains in 333 complexes that contained 59,559 residue contacts. In the assessment of ISIS, no sequence that was used for training had any significant similarity for any of the sequences that were used for testing. That is, no protein in the test set could have been modeled by any protein in the development sets by homology-based predictions [43,44].

### Implementing and applying other methods.

We chose methods that represent the variety of approaches for predicting interaction sites. ProMate [26] is a structure-based method that extracts features from an unbound chain and uses them to predict the binding site. We also chose three sequence-based methods: a sequence-only method [28], an evolutionary-based method (ET [35,45]), and a biophysics-based method (hydrophobic moment [22]). The first two were available as servers for public use. The hydrophobic moment was not publicly available; thus, we implemented it for the purpose of this analysis. We chose an operating point of coverage equal to 15%, which was the highest coverage reached by the hydrophobic moment tool.

### Comparing hotspots with other interface residues.

We used the dataset of interface residues that was used to test ISIS originally [28]. In this dataset there are more than 20,000 interface residues, 2,182 of which were classified by ISIS as positive. Attempting to zoom in on the differences between hotspots and other interface residues, we compared the features of these 2,182 residues with the features of the residues that were classified as negative. The results of the comparison for amino acids are presented in Table 1, and are based on the evolutionary profile we used for prediction. For each interface residue, we used a multiple sequence alignment to check how often each residue is present in this position. We performed the same analysis for all the positions that were found experimentally, by alanine scanning, to be hotspots. Table 1 shows the average percentage occupancy of each amino acid in all positively predicted positions in all negatively predicted interface residues.

## Abbreviations

ET: evolutionary trace
PDB: Protein Data Bank
